# Host cytoskeleton and membrane network remodeling in the regulation of viral replication

**DOI:** 10.52601/bpr.2024.240040

**Published:** 2025-02-28

**Authors:** Xuedi Gao, Xinming Chen, Letian Yu, Shuangshuang Zhao, Yaming Jiu

**Affiliations:** 1 Unit of Cell Biology and Imaging Study of Pathogen Host Interaction, The Center for Microbes, Development and Health, Key Laboratory of Molecular Virology and Immunology, Shanghai Institute of Immunity and Infection, Chinese Academy of Sciences, Shanghai 200031, China; 2 University of Chinese Academy of Sciences, Beijing 100049, China; 3 Key Laboratory of Virology and Biosafety, Chinese Academy of Sciences, Wuhan 430071, China

**Keywords:** Viral replication factory Cytoskeleton, Actin filaments, Microtubules, Intermediate filaments, Rearrangement of cellular membranes

## Abstract

Viral epidemics pose major threats to global health and economies. A hallmark of viral infection is the reshaping of host cell membranes and cytoskeletons to form organelle-like structures, known as viral factories, which support viral genome replication. Viral infection in many cases induces the cytoskeletal network to form cage-like structures around viral factories, including actin rings, microtubule cages, and intermediate filament cages. Viruses hijack various organelles to create these replication factories, such as viroplasms, spherules, double-membrane vesicles, tubes, and nuclear viral factories. This review specifically examines the roles of cytoskeletal elements and the endomembrane system in material transport, structural support, and biochemical regulation during viral factory formation. Furthermore, we discuss the broader implications of these interactions for viral replication and highlight potential future research directions.

## INTRODUCTION

Viruses are obligate intracellular parasites that rely on the host cell machinery for their reproduction (Eisenreich *et al.* 2019). The viral life cycle can be divided into four major stages: entry, replication, assembly, and release. First, the virus enters the host cell by recognizing and binding to specific surface receptors. The viral genome is then released and used as a template for replication and transcription. Newly synthesized viral components are subsequently assembled into viral particles, which are finally transported and released from the cell.

Viral infection alters the spatial distribution and biological functions of intracellular organelles and macromolecules, with particularly prominent changes to the cytoskeleton and membrane systems. This review summarizes the morphological changes and biological functions of the cytoskeleton and membrane systems during viral replication. Additionally, it highlights the similarities and differences in replication strategies across various viruses, suggesting potential avenues for broad-spectrum antiviral drug development.

## THE CYTOSKELETON AND ENDOMEMBRANE SYSTEM

**Figure 1:**
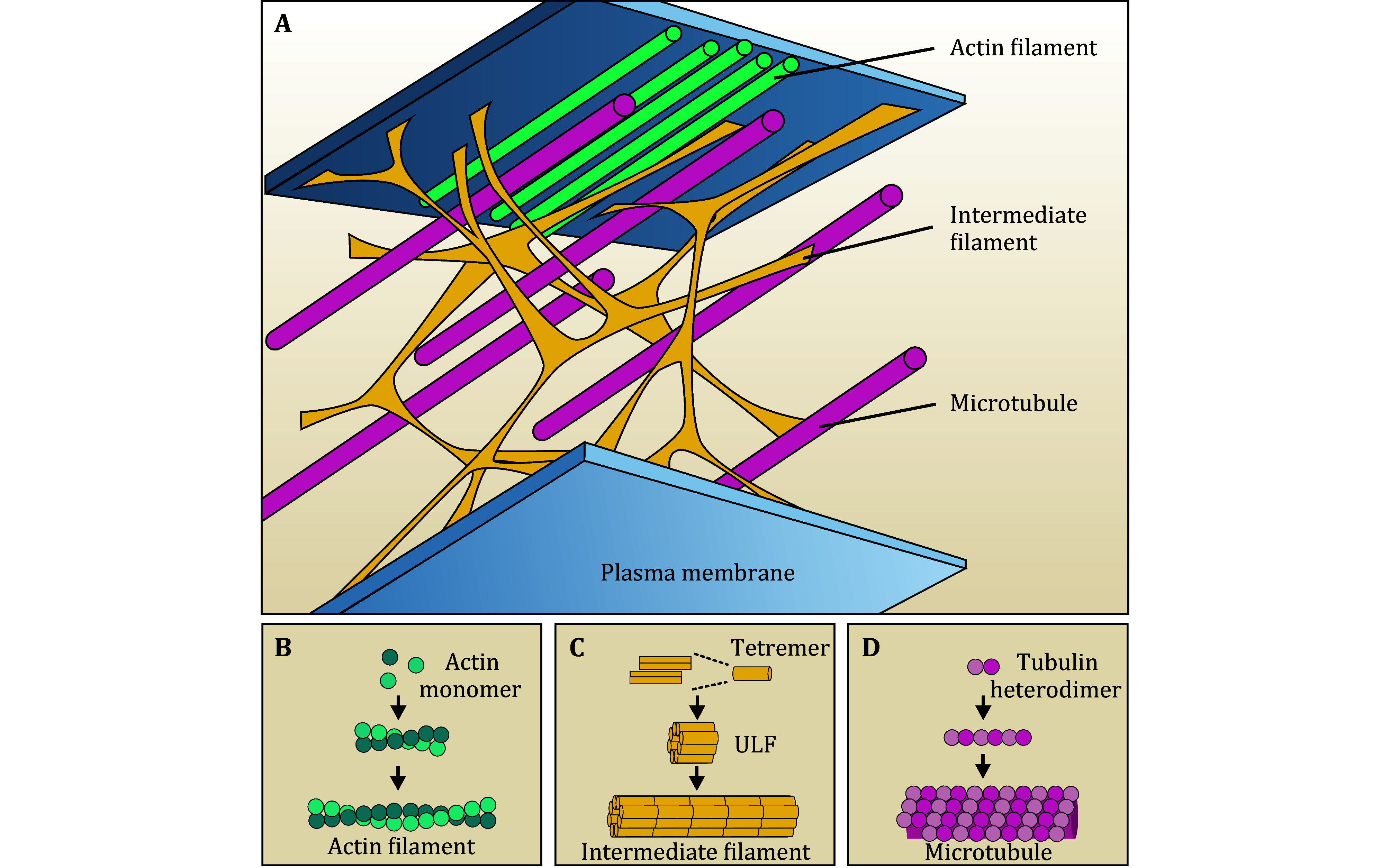
Morphology and assembly of the cytoskeleton. **A** Cellular distribution of actin filaments, microtubules, and intermediate filaments. **B** G-actin monomer is gradually assembled into F-actin. **C** The monomers of intermediate filaments are gradually assembled into a tetramer, unit-length filament (ULF) and mature filament. **D** α- and β-tubulin monomers are dimerized to form tubulin heterodimer, which then is assembled into hollow tubular structures with a diameter of about 25 nm

The cytoskeleton is a critical component of eukaryotic cells and is highly dynamic, with the architecture and intracellular distribution altering through filament assembly and disassembly. The cytoskeleton primarily consists of three fundamental networks: actin filaments, microtubules, and intermediate filaments, along with their associated proteins (Fig. 1A). Actin filaments are formed from globular actin (G-actin) monomers, resulting in filamentous actin (F-actin) with an approximate diameter of 7 nm (Fig. 1B) (Svitkina 2018). Microtubules are hollow structures approximately 25 nm in diameter, formed by the polymerization of α-tubulin and β-tubulin monomers, and radiate from the microtubule organizing center (MTOC) to the cell periphery (Figs. 1A and 1D). Various actin-binding proteins and microtubule-associated proteins regulate the assembly and disassembly of cytoskeletal filaments, while motor proteins like kinesin, dynein, and myosin coordinate cargo transport and organelle distribution (Mizuno *et al.* 2004). Unlike actin filaments and microtubules, intermediate filaments exhibit tissue-specific expressions. They share a common secondary structure, with diameters of approximately 10 nm, extending from the nucleus to the cell periphery (Fig. 1A). Intermediate filaments gradually assemble from monomers into dimers, tetramers, unit-length filaments (ULF), and mature filaments (Fig. 1C). Compared to the other cytoskeletal networks, intermediate filaments are more resilient and serve as vital mechanical support for the cell (Herrmann *et al.* 2007). Overall, the cytoskeleton is crucial for maintaining cell morphology and the organization of internal structures, while also participating in key cellular processes, including intracellular transport, signal transduction, cell division, and pathogen infection (Gong *et al.* 2022; Zhang *et al.* 2021).

The endomembrane system of eukaryotic cells consists of several biological membranes, including the endoplasmic reticulum, Golgi apparatus, secretory vesicles, lysosomes, nuclear membrane, endosomes and plasma membrane. Continuity exists between these membranous organelles because of structural links or the occurrence of membrane fusion. The continuous movement of the endomembrane system needs the involvement of the cytoskeleton and cytoskeleton-based motor proteins (Brandizzi and Wasteneys 2013; Wang and Hussey 2015). The endomembrane system is essential for various cell functions, including protein synthesis, lipid synthesis, energy production, signal transduction, as well as cellular phagocytosis and excretion (Simpson 2020). The highly dynamic nature and functional coordination of the endomembrane system ensures normal cell growth, division, and the execution of its functions. During infection, the virus hijacks host cell components and alters the normal morphology and biological functions of the intracellular membrane system to facilitate the efficient progression of its life cycle.

## VIRAL REPLICATION INDUCES STRUCTURAL REMODELING IN CELLULAR NETWORKS

Viruses induce substantial reorganization of the host cytoskeleton and membrane compartments, forming organelle-like inclusions for viral replication and assembly, known as viral factories (de Castro *et al.* 2013). These structures incorporate host proteins and viral components essential for replication, physically isolating the replication site from the rest of the intracellular environment, thereby enhancing the efficiency of viral replication and assembly. Viral factories can form in either the cytoplasm or the nucleus, depending on the type of virus (Netherton and Wileman 2011).

### Classification of viral factory

**Figure 2:**
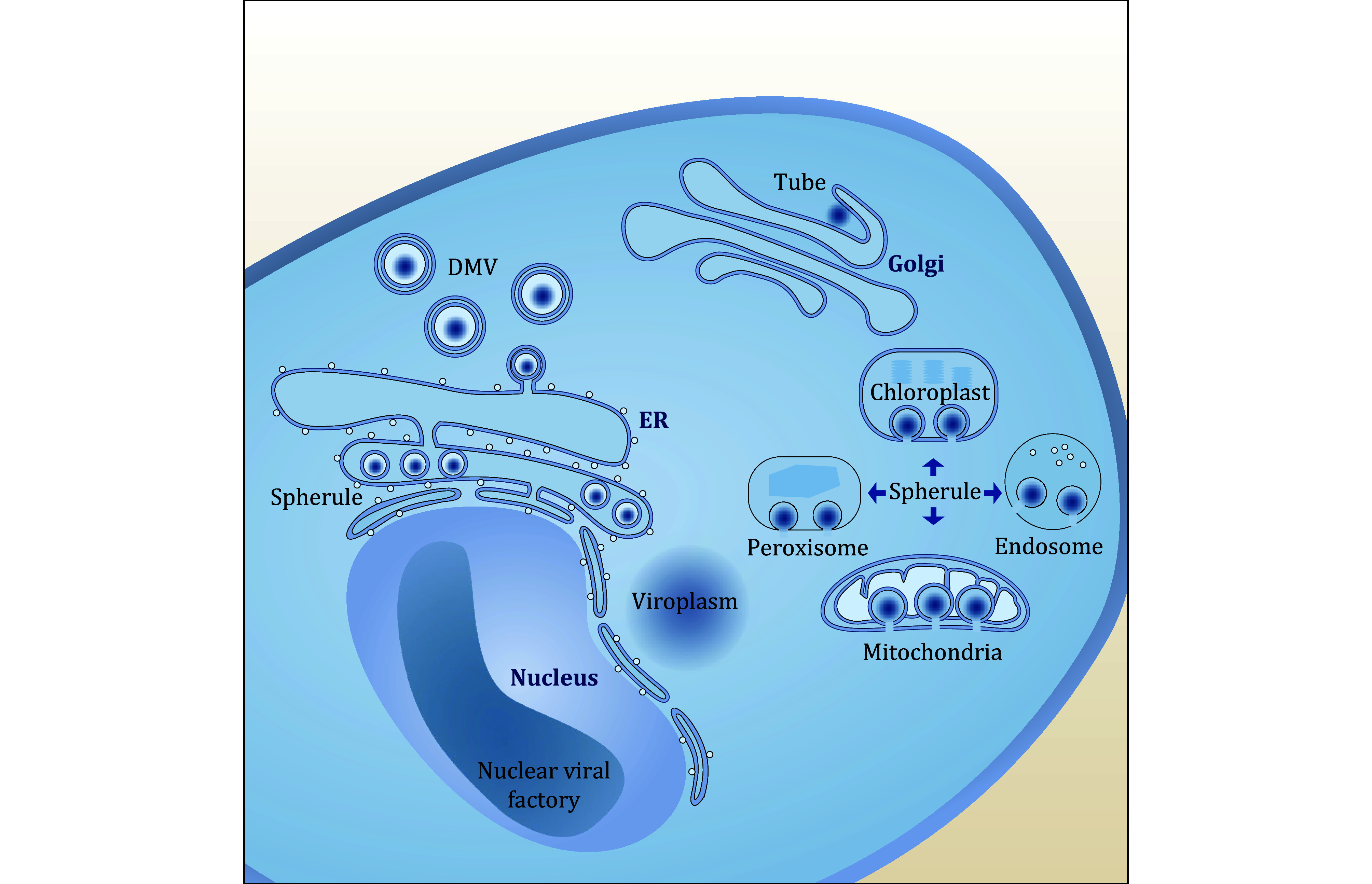
Types of viral factory. According to the differences in morphology and structure, cytoplasmic viral factories can be divided into four categories, including viroplasm, spherule, DMV and tube. Spherules can originate from mitochondria, peroxisome, chloroplast, endosome and endoplasmic reticulum. DMVs originate from the endoplasmic reticulum, and tubes originate from Golgi. Nuclear viral factories exist in the dorsal side of cellular nucleus

The structure of viral factories varies depending on the virus and host cell type, and can be classified into four major categories based on morphological and structural differences (Fig. 2): viroplasm, spherule, double membrane vesicle (DMV), and tube.

Viroplasm is an electron-dense intracellular cytoplasmic inclusion for viral replication and assembly, formed by nucleo-cytoplasmic large DNA viruses (NCLDV) like *Poxviridae* (Laliberte and Moss 2010), *Asfarviridae* (Andrés *et al.* 1998), *Iridoviridae* (Netherton *et al.* 2007), double strand RNA viruses (dsRNA) like *Reoviridae* (Touris-Otero *et al.* 2004), and negative-sense single-stranded RNA viruses (ssRNA(-) virus) like *Filoviridae* (Dolnik *et al.* 2015).

Spherule is intramembrane depression with a diameter ranging from 50 to 400 nm that facilitates viral replication. Depending on the type of virus, it can appear on different organelle membrane components. For instance, on chloroplasts in *Tymovirus* infections, peroxisomes in *Tombusvirus* infections, mitochondria in *Nodaviridae* infections, endosomes and lysosomes in *Togaviridae* infections, and the endoplasmic reticulum in *Flaviviridae* infections (Diaz and Ahlquist 2012).

DMV is a double-layered membranous structure that originates in the endoplasmic reticulum or Golgi apparatus, with diameters ranging from 200 to 300 nm. Infections with *Picornaviridae*, *Coronaviridae*, and *Arterividae* all result in intracellular DMV structures (Wolff *et al.* 2020). A common feature of viruses that produce spherules and DMVs is the presence of double-stranded RNA (dsRNA) replication intermediates. Replication of dsRNA in isolated membrane compartments helps evade detection by the host’s innate immune system.

Tube is a tubular membrane structure that originates from the cellular Golgi apparatus and has a diameter of around 100 to 150 nm. It is the site of intracellular replication for *Bunyaviridae* viruses (Fontana *et al.* 2008). Tubes typically have a spherical head of 120 to 150 nm and a cylindrical rod measuring 80 to 100 nm in diameter, which opens into the cytoplasm and includes viral dsRNA, viral proteins, and host cell proteins for viral replication.

Nuclear viral factories are formed by viruses that replicate within the nucleus, inducing significant structural remodeling similar to that seen in cytoplasmic viral factories. For instance, the Herpes simplex virus creates replication compartments (RCs) in the nucleus, for viral DNA replication and late gene transcription (Chang *et al.* 2011). Baculovirus infections induce the formation of subnuclear structures within the nucleus, including virogenic stroma (VS) and peristromal region (PR), which provide a molecular scaffold for viral DNA replication and assembly (Nagamine *et al.* 2008; Rohrmann 2013).

Viral factories are dynamic structures that assemble and disassemble specific components throughout different stages of the viral life cycle. They exploit intracellular materials and signaling pathways to stay connected with cellular transport routes, integrate necessary components, and facilitate the exit of newly formed viral particles (Fig. 2).

### Remodeling of the endomembrane system during viral infection

**Figure 3:**
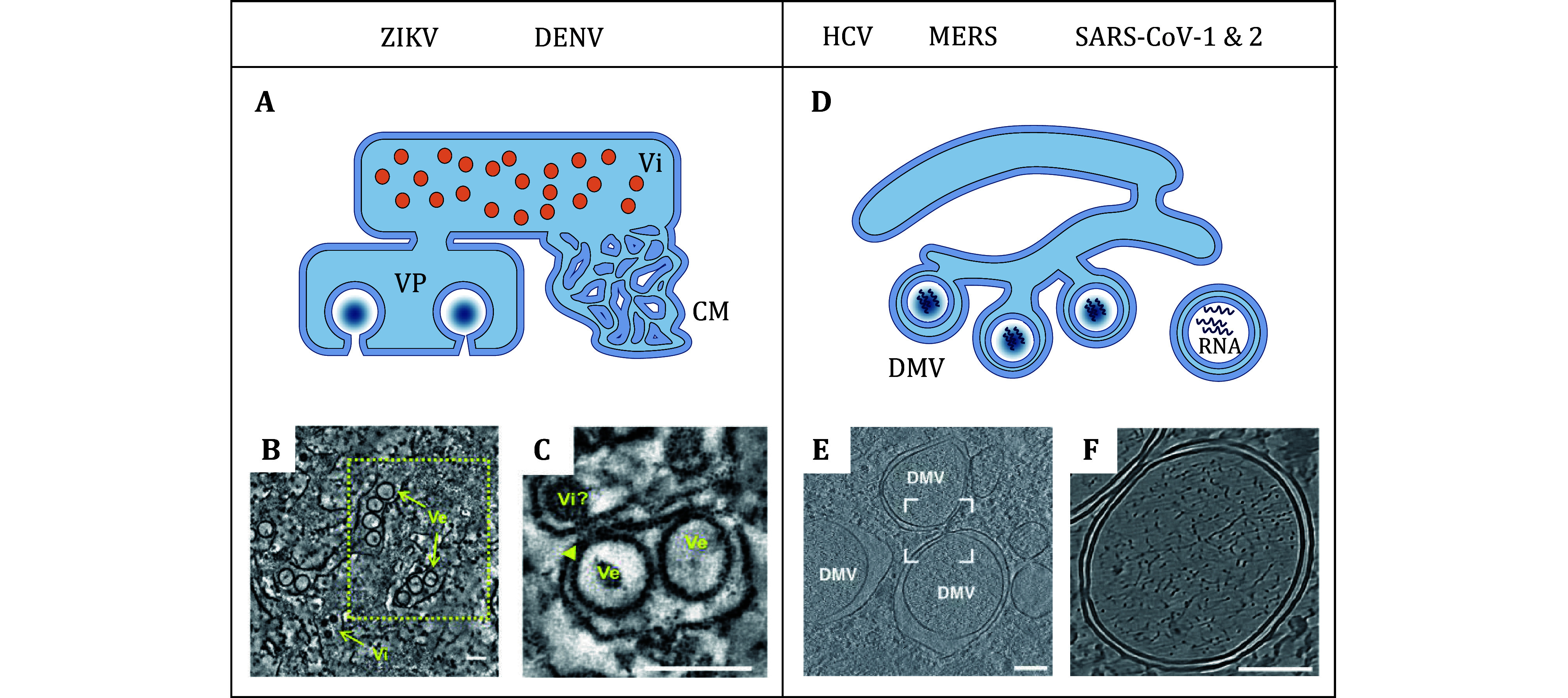
Spherical viral factories originating from the endoplasmic reticulum. **A** Schematic representation of the Flavivirus (ZIKV, DENV) replication and assembly compartments. Genome replication occurs within VPs formed upon ER membrane invagination. Vi: virions. **B** Electron tomography of ZIKV-induced VPs in hNPCs. A slice through a tomogram is shown depicting ZIKV-induced vesicles (Ve) within the rough ER as well as Vi. Scale bar: 100 nm (adapted from ref. (Cortese *et al.* 2017) with copyright permission from Creative Commons license). **C** Slice through the tomogram showing the pore-like openings (colored arrowheads) of ZIKV induced vesicles toward the cytoplasm. A potential ZIKV budding event (Vi) on the ER tubule opposing the vesicle pore can be observed. Scale bar: 100 nm (adapted from ref. (Cortese *et al.* 2017) with copyright permission from Creative Commons license). **D** Schematic representation of DMVs formed by ER protrusions. Typical viruses include HCV, MERS-CoV, SARS-CoV-1, SARS-CoV-2. DMVs are responsible for viral genome replication. **E** Tomogram showing DMVs in VeroE6 cells infected with SARS-CoV-2 at 16 hpi. Scale bar: 100 nm (adapted from ref. (Klein *et al.* 2020) with copyright permission from Creative Commons license). **F** Tomogram showing DMVs and RNA filaments in A549-ACE2 cell infected with SARS-CoV-2. Scale bar: 100 nm (adapted from ref. (Klein *et al.* 2020) with copyright permission from Creative Commons license)

In this part, certain well-known pathogenic viruses are used as examples. The classical forms of viral factories and their variants in host cytoskeletal networks are discussed in detail. Almost all RNA viruses create viral factories in the cytoplasm, which are closely linked to host membrane components. For instance, Dengue virus (DENV) and Zika virus (ZIKV) exploit the host rough endoplasmic reticulum to generate vesicle packets (VPs) approximately 90 nm in diameter for viral genome replication, with pore-like openings of around 11 nm allowing material exchange with the cytoplasm (Figs. 3A–3C) (Cortese *et al.* 2017; Welsch *et al.* 2009). Additionally, the smooth endoplasmic reticulum, located near the mitochondria and VPs, generates convoluted membranes (CMs) (Fig. 3A), which are rich in viral proteins and may serve as sites for viral polyprotein maturation (Neufeldt *et al.* 2018).

Hepatitis C virus (HCV) from the Flaviviridae family, Middle East respiratory syndrome-related coronavirus (MERS-CoV), severe acute respiratory syndrome coronavirus 1 (SARS-CoV-1), and SARS-CoV-2 from recent outbreaks all use the host endoplasmic reticulum to form clusters of outwardly protruding double-membrane vesicles (Fig. 3) (Klein *et al.* 2020; Neufeldt *et al.* 2018; Paul *et al.* 2013; Wolff *et al.* 2020). The space between the inner and outer membranes of the DMV derives from the ER lumen, and the area inside the inner membrane is rich in viral RNA (Figs. 3D–3F). This environment enhances viral RNA replication while also allowing the virus to evade immune detection (Tabata *et al.* 2021). In the later phases of viral infection, the outer membranes of the DMVs fuse with the endoplasmic reticulum membranes, preparing for viral budding (Wen *et al.* 2020).

The similarities and differences in the morphology and origin of viral factories in different virus categories are determined by the types of organelles utilized for viral genome replication and viral protein synthesis. During viral infection, organelles have a markedly different shape and spatial distribution than healthy cells. While studies are continuing to reveal the true nature of these fine structures using advanced technology, it is unclear how viruses regulate such complexes and the detailed structural changes within the cell. Moreover, further research is needed to uncover the systematic molecular mechanisms underlying these processes.

## REMODELING OF THE CYTOSKELETON NETWORK DURING VIRAL INFECTION

**Figure 4:**
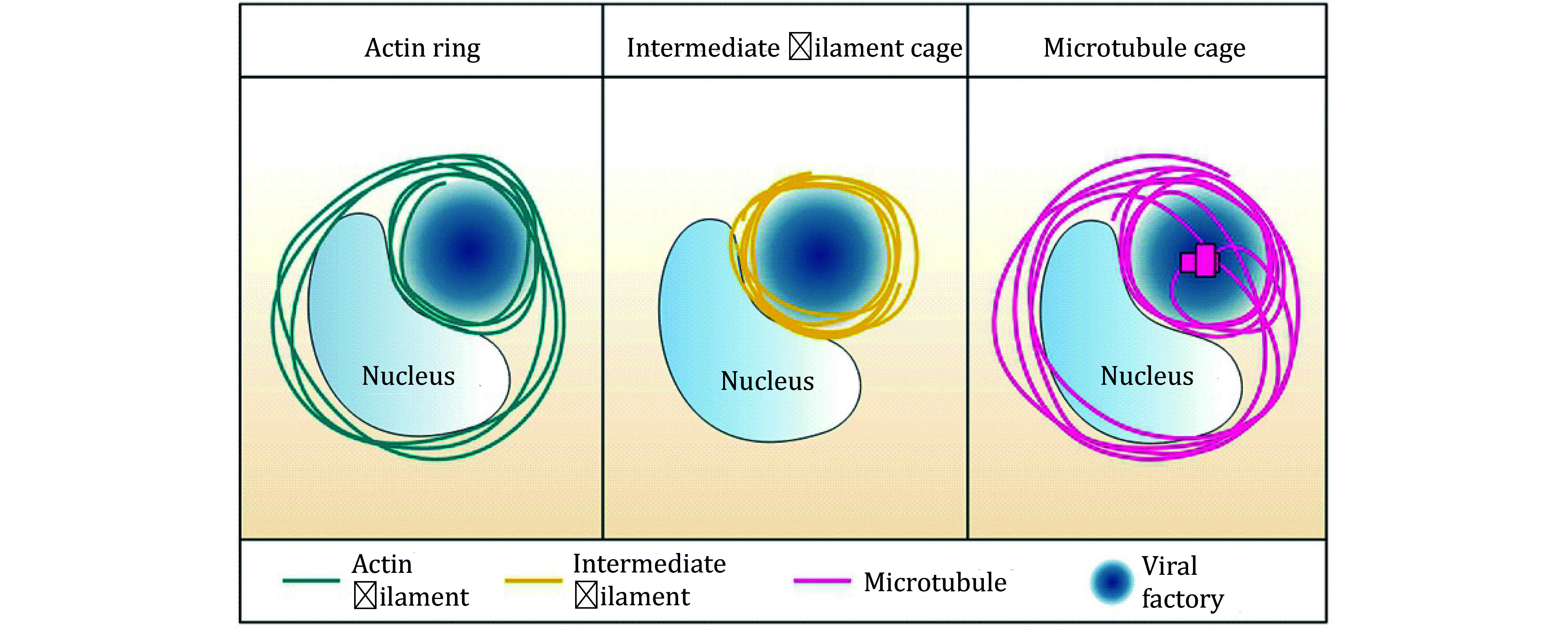
Viral infection induces cytoskeleton remodeling into cage-like structure. Virus infection can cause severe rearrangement of the cytoskeleton, and all three kinds of cytoskeleton form a cage-like structure enclosing the near-nuclear virus factory. Microfilaments form actin ring around the virus components, endoplasmic reticulum, Golgi apparatus, etc.; intermediate filaments form an intermediate filament cage to completely wrap the virus factory; microtubules form a grid shape microtubule cage

During the formation of viral factories, not only does the endomembrane system undergo substantial reorganization, but the structure of the cytoskeleton also experiences significant changes. Various viral infections can induce the formation of cage-like structures around viral factories, including actin rings, microtubule cages, and intermediate filament cages (Cortese *et al.* 2017; Wen *et al.* 2020; Zhang *et al.* 2022) (Fig. 4).

For instance, in cells infected with porcine epidemic diarrhea virus (PEDV) and transmissible gastroenteritis virus (TGEV), actin filaments contract from the cell periphery to the perinuclear region, forming a ring-like structure that encircles viral particles near the nuclear membrane, thereby assisting in replication and assembly (Wang *et al.* 2009). Similarly, in recent global outbreaks of SARS-CoV-2 infection, actin rearranges to form a ring-like structure that encases the replication organelles. The formation of this actin ring helps concentrate factors necessary for the synthesis of progeny viruses, enclosing them around the replication organelles and thereby facilitating the viral replication process (Kloc *et al.* 2022) (Fig. 4).

Current studies have discovered a variety of RNA and DNA viruses that can induce intermediate filaments to rearrange (Zhang *et al.* 2020). For instance, during dengue and Zika virus infections, intermediate filaments protein vimentin and nestin dynamically rearrange to form cage-like structures that contain perinuclear viral factories, facilitating viral replication (Cortese *et al.* 2017; Teo and Chu 2014; Zhang *et al.* 2022). During the viral DNA replication and expression stages of the African swine fever virus (ASFV), Calcium/Calmodulin-dependent protein kinase II (CaM kinase II) phosphorylates vimentin and transports it to the viral assembly site at the edge of the replication factory, providing structural support (Stefanovic *et al.* 2005). Six to nine hours after SARS-CoV-2 infection, vimentin filaments undergo conformational changes to form a “cage” that encapsulates clusters of DMVs (Cortese *et al.* 2020) (Fig. 4).

In contrast to vimentin, reports of microtubule cages are relatively rare. Through live-cell imaging and 3D reconstruction, only one study found that within 20 hours post-Zika virus infection, microtubules begin to reposition and form a distinct cage-like structure, enclosing viral dsRNA, NS3, and the reorganized endoplasmic reticulum (Cortese *et al.* 2017) (Fig. 4).

Viral infection also leads to morphological changes in the cytoskeleton in the nucleus. For instance, human cytomegalovirus (HCMV) infection remodels the nucleus shape and changes the spatial distribution of nuclear material. As the infection continues, the nuclear actin filaments transit from a diffuse pattern to short rods and subsequently form a filamentous network, before progressively reverting to a diffuse form in the later stages (Procter *et al.* 2020). Nevertheless, the role of the highly dynamic nuclear cytoskeleton in viral replication remains to be further investigated.

## FUNCTIONS OF THE CYTOSKELETON AND ENDOMEMBRANE SYSTEMS IN VIRAL REPLICATION

The cytoskeletal network is frequently recruited to interact with restructured membranes, aiding in the formation and maintenance of viral replication factories. As mentioned, viruses hijack host organelles, primarily the endoplasmic reticulum and Golgi, to form replication factories where viral and host factors aggregate for genome replication and protein synthesis (Fig. 3). These chambers shield the viral genome from immune detection, with a small opening enabling the release of new viral particles for maturation.

### Transport function of the endomembrane system

Apart from forming viral replication factories, the host’s endocytic recycling compartments (ERC) are involved in the intracellular viral transport. Specifically, viruses utilize endosomes, such as those formed by clathrin or caveolin, to facilitate their entry into host cells (Nicola *et al.* 2013). Additionally, endosomes provide a finely tuned environment for virus endocytosis. For instance, simian virus 40 (SV40) enters early endosomes, late endosomes, and endolysosomes before reaching the endoplasmic reticulum, with endosome acidification being critical for infection (Engel *et al.* 2011). Once internalized, viruses often modify vesicle sorting mechanisms to evade degradation by the host’s cellular machinery. They interact with and hijack cellular trafficking proteins to mislocalize immune regulators and create platforms for viral assembly (Cruz and Buchkovich 2017). Finally, the endomembrane system is also integral to the egress of viruses from host cells. During the budding process, viruses exploit cellular pathways, including those involving the endocytic recycling compartment, to facilitate the release of newly formed virions. For example, viruses can hijack the Rab11 pathway to promote efficient budding and release, ensuring that the viral particles can escape and infect neighboring cells (Vale-Costa and Amorim 2016).

### Transport function of the cytoskeleton

The cytoskeleton facilitates viral transport to replication sites. Actin filaments predominantly assist the movement of viruses to the cell periphery after entry, while polarized microtubules and their associated motor proteins are required for directed viral transport to nuclear or perinuclear replication sites. For instance, Influenza A virus (IAV) initially travels along actin filaments at the cell periphery and then moves through the cytoplasm along microtubules to reach the perinuclear region for genome release (Zhang *et al.* 2018). Within the host cell, motor proteins myosin VI and dynein work together to transport viral vesicles along actin filaments and microtubules. Notably, the ‘driver switch' mechanism from actin filaments to microtubules enables the successful spread of the virus within the host cell (Zhang *et al.* 2018).

The cytoskeleton not only helps in viral movement but also in the fusion of viral factories. Early in the infection, smaller viral factories move along the microtubules in an undirected manner. As the infection progresses, microtubules rearrange, leading to the contraction of the host cell’s cytoplasm and membrane. Ultimately, as the host cell shrinks and fuses to form larger viral replication factories, smaller viral factories distributed along the microtubules cease their movement and approach the host cell’s proximal nucleus (Schramm *et al.* 2006). Viral replication and assembly are enhanced as viruses utilize dynamic microtubules to build larger viral factories in the perinuclear region.

The cytoskeleton and intracellular membrane system work synergistically to facilitate viral transport and intercellular spread (He *et al.* 2023; Reichel *et al.* 1999). For example, following RNA synthesis, influenza virus vRNPs are released from the nucleus, accumulate at microtubule-organizing centers (MTOCs), and rely on microtubule networks to reach the plasma membrane, where they interact with vesicular trafficking systems for release (Simpson and Yamauchi 2020). Similarly, during HIV-1 infection, actin filaments not only guide the viral replication and transcription complex (RTC) but also coordinate with endosomal membranes to transport reverse transcription products from the nucleus to the cytoplasm (Ospina Stella and Turville 2018; Rey *et al.* 1996). This coordinated transport involves dynamic interactions among viral capsid proteins, endosomal membranes, actin filaments, and motor proteins, ensuring efficient intracellular trafficking and subsequent viral release.

The dynamic nature of the cytoskeleton is essential for the formation of viral factories, and disrupting cytoskeletal dynamics with depolymerizing or stabilizing drugs significantly impairs viral replication. For instance, the actin filament-stabilizing drug Jasplakinolide alters the structure of Bunyavirus viral tubes, shifts the location of viral assembly sites, and reduces the release of infectious viral particles into the culture supernatant (Fontana *et al.* 2008). The microtubule-stabilizing drug paclitaxel significantly decreases the viral yield of Zika virus and tick-borne encephalitis virus (TBEV) (Bílý *et al.* 2015; Cortese *et al.* 2017). Additionally, the intermediate filament-disrupting drug Withaferin A (WFA) markedly reduces SARS-CoV-2 replication (Cortese *et al.* 2020). Together, the normal structure and physiological function of the cytoskeleton are crucial for the efficiency and precision of viral infection.

### Biochemical functions of cytoskeletal proteins

In many viral infections, host cytoskeletal proteins interact with viral proteins to promote viral replication. Proteomic investigations have shown that many backbone proteins are involved in the replication of the Ebola virus (EBOV) (Muthaiyan *et al.* 2020; Spurgers *et al.* 2010). The host proteins that interact with the polymerase L protein of EBOV include cytoskeletal remodeling molecules such as CNN3, FLNA, DOCK7, CTTN, CORO7, and AMOT (Fang *et al.* 2022). Among them, the VP35 protein of EBOV interacts with the light chain of the microtubule motor protein dynein (DLC8), thereby enhancing viral RNA replication (Kubota *et al.* 2009; Luthra *et al.* 2015). EBOV’s VP40 protein interacts with the cellular IQGAP1 protein to hijack and rearrange the cytoskeleton, facilitating the viral budding and release (Lu *et al.* 2013). Similarly, mass spectrometry has revealed that CCHFV’s NP protein interacts with actin, tubulin, and vimentin to promote viral replication (Surtees *et al.* 2016). Additionally, certain multifunctional molecules, such as the microtubule-binding protein STAU1, bind to both the cytoskeleton and viral RNA, coordinating viral components with host factors to enhance efficient viral RNA replication (Fang *et al.* 2018).

Furthermore, the cytoskeleton has the ability to directly stimulate the transcription of the viral DNA by acting as a transcription factor. For instance, actin filaments alter the structure of the ribonucleoprotein (RNP) of parainfluenza virus type 3 (HPIV3), converting it from a loosely coiled state to a relatively compact structure (Gupta *et al.* 1998). This structural change facilitates the binding of actin filaments to viral RNA polymerase or cis-acting transcription elements, thereby promoting the transcription and translation of the viral genome (Gupta *et al.* 1998). Disruption of actin filaments impedes viral RNA synthesis and RNP accumulation, leading to impaired viral replication (Gupta *et al.* 1998). Actin binds to the respiratory syncytial virus (RSV) genomic template in a divalent cation-dependent manner. As a classical viral transcription factor, actin works together with the host transcriptional cofactor profilin to regulate the transcription of the viral genome (Burke *et al.* 2000).

### Structural support of the cytoskeleton

Viral replication factory structures are formed and maintained in part by cytoskeletal proteins, which not only envelop the viral factories but also actively participate in their development. For instance, actin filaments and microtubules cooperate to enhance the rotavirus (RV) viroplasm’s assembly and production process (Vetter *et al.* 2022). Furthermore, actin aids the Bunyavirus in establishing a tube viral factory within the cellular Golgi apparatus (Fontana *et al.* 2008). Using electron microscopy, a fibrous texture structure was observed inside the tubes. When paired with matrix aided peptide mass fingerprinting by matrix assisted laser desorption-ionization mass spectrometry (MALDI-MS), it was discovered that the purified tubes included actin, tubulin, and myosin, three essential cytoskeletal proteins. Furthermore, actin is associated with intracellular immature viruses, indicating that actin is involved in both the morphogenesis of viruses in the Golgi membrane and the formation of tubes (Fontana *et al.* 2008).

The cytoskeleton aids in anchoring the viral factory to its host organelle. The formation and function of Bunyavirus tubes are facilitated by Golgi-associated cytoskeletal proteins, which also serve to anchor the tubes to the Golgi membrane and preserve the structural integrity of the viral factory (Fontana *et al.* 2008).

In our previous study (Zhang *et al.* 2022), we discovered that after ZIKV infection, cellular vimentin filaments gradually aggregate in the perinuclear region to form a cage-like structure that encapsulates the viral replication factory, converges important host factors and viral components, and interacts with a variety of endoplasmic reticulum proteins, including ribosome-binding protein 1 (RRBP1), to facilitate viral RNA replication. When cells were depleted of vimentin, the ZIKV factory exhibited intracellular dispersion, with lower levels of viral genome replication, viral protein synthesis, and mature virions generation. The discovery that vimentin filaments help to form complete and dense intracellular viral replication factories for viral production in both physical space and biological regulation suggests that vimentin could be a potential host target for ZIKV infection treatment.

## TECHNIQUES TO EXPLORE INTERACTIONS BETWEEN VIRAL FACTORIES AND THE CYTOSKELETON

### Optical microscopy imaging

Study on the relationship between viral factories and the cytoskeleton involves describing their structure, biogenesis, and function over the progress of infection. Using fluorescent probes and super-resolution microscopy (such as STED, SIM, and single-molecule imaging like dSTORM, PALM, PAINT, and ROSE) reveals the macromolecular structure and localization within viral factories. For instance, STED is well-suited for super-resolution live-cell imaging; it has been used to visualize the delivery of adenoviral DNA and the entry of pseudotyped HIV particles (Witte *et al.* 2018). Single-molecule imaging overcomes the diffraction limit of light, achieving resolutions down to 20 nm, and providing insights into viral assembly. For example, dSTORM has enabled detailed structural analysis of HSV-1, revealing the spatial organization of tegument and envelope proteins (Laine *et al.* 2015). Collectively, these advanced imaging approaches allow us to map dynamic processes with unprecedented precision, uncovering key molecular interactions essential for viral replication and assembly.

Additionally, these imaging techniques that overcome the optical diffraction limit enable nanoscale live-cell imaging, enabling dynamic tracking of the viral factory assembly process and host components, such as the cytoskeleton, as they accumulate around the viral factories and undergo morphological reconfiguration to form enclosures (Zhang *et al.* 2022). Employing convolutional neural network-based automated cell classification and analysis pipelines allows for the extraction of image features, enabling automated analysis, classification, and measurement of large volumes of cellular morphology data (Procter *et al.* 2020). Sparse deconvolution is then applied to improve the spatiotemporal resolution of live cell pictures (Zhao *et al.* 2022).

Probes that image individual viral RNA molecules are effective tools for analyzing viral replication and assembly. For instance, fluorescence in situ hybridization (FISH), reveals the viral genome’s localization as well as the location of assemblies that generate new viral particles. The recently discovered live FISH technology (Wang *et al.* 2019) for imaging DNA and RNA in living cells has the potential to be used to study viral factories by dynamically exhibiting viral gene assembly, transport, and particle assembly.

### Electron microscopy imaging

Apart from optical microscopy, electron microscopy plays an important role in studying the structure of viral factories. Transmission electron microscopy (TEM) is used to examine ultra-thin sections of infected cells (Roingeard 2008). Correlative light and electron microscopy (CLEM) allows for precise, high-resolution imaging of subcellular organelle regions of interest. Immunogold labeling experiments employ cryo-electron microscopy to determine the spatial connection between target proteins and viral replication factory structures. Imaging followed by electron tomography (ET) and 3D reconstruction provides a better and more intuitive presentation of the results (Peddie *et al.* 2022; Romero-Brey 2018).

### Molecular biology technology

Various microscopy techniques can reveal the timing, intracellular location, and fine structure of viral factory formation, while some molecular and biochemical virology experiments can provide deeper insights into their molecular mechanisms. For instance, biochemical assays that detect protein-protein interactions, such as co-immunoprecipitation, yeast two-hybrid (Y2H), and proximity-dependent biotin identification (Bio-ID), can capture cytoskeletal components and related proteins interacting with viral proteins during the early stages of infection. CRISPR-Cas9 gene editing can be employed to construct stable cell lines by targeting and knocking out specific genes, or combined with cytoskeletal-targeting drugs, such as the actin filament polymerization inhibitor cytochalasin D, the microtubule polymerization inhibitor nocodazole, or the intermediate filament disruptor withaferin A (WFA). Additionally, subsequent molecular virology techniques, such as real-time quantitative PCR (RT-PCR) to measure viral genome replication levels, Western blot (WB) to assess viral protein levels, and titration assays to quantify infectious viral particles secreted, can qualitatively and quantitatively assess the impact of cytoskeletal components and their dynamics and stability on the formation of viral replication factories and viral yield.

## SUMMARY AND OUTLOOK

Viral replication requires precise regulation of signaling pathways and cellular structures to balance host cell survival with efficient viral replication. The restructuring of the cytoskeleton and endomembrane system induced by viral infection creates a unique site for viral replication—the viral factory. With ongoing advancements in technology, significant progress has been made in this field, offering new insights into the cell biology of viral infections and viral evolution. However, our understanding remains incomplete regarding the associated cytoskeletal rearrangements, membrane morphology changes, dynamic processes of viral factory formation, biological functions, regulatory mechanisms, and interrelationships among these factors, despite researchers uncovering some aspects of the composition and fine structure of viral factories.

Viruses rely on and reshape cytoskeletal structures to invade, replicate, transport, assemble, and release. Investigating the role of the cytoskeleton in infections caused by highly pathogenic viruses across various cell types may help disrupt viral replication at different stages, thereby achieving control and prevention. Moreover, as the cytoskeleton is an essential structure in eukaryotic cells, it helps to identify broader mechanisms and molecular targets for viral replication. Several directions are worth exploring in the future: (1) The spatiotemporal relationship between cytoskeletal and endomembrane system remodeling during viral infection. Current studies focus on the involvement of the cytoskeleton in endomembrane transport and morphology maintenance under normal conditions, but the mechanisms during infection remain underexplored. (2) The interaction sites between the cytoskeleton and viral components. By comparing the similarities and differences in the interaction sites of different viruses with the same cytoskeletal protein, we can uncover general mechanisms of pathogen-host interactions and design targeted drugs to develop broad-spectrum antiviral therapies. (3) The relationship between differences in cytoskeletal components across different cells and the tissue-specificity of viral infections. Cells contain various cytoskeletal proteins, particularly intermediate filament proteins, which are tissue specific. Disruption of different types of cytoskeletal structures may lead to different diseases and relate to the specificity of viral infections.

In summary, the cytoskeleton plays a direct role in viral factory formation. In addition to assembling the cytoskeleton into a filamentous network that acts as a physical support and material transport track, cytoskeletal proteins are multifunctional and participate in a variety of bioregulatory processes that carry out biochemical activities. Investigating how the cytoskeleton interacts with viruses will help us understand the molecular aspects of viral replication and identify potential therapy targets.

## Conflict of interest

Xuedi Gao, Xinming Chen, Letian Yu, Shuangshuang Zhao and Yaming Jiu declare that they have no conflict of interest.
